# Role of melatonin in embryo fetal development

**Published:** 2014

**Authors:** SE Voiculescu, N Zygouropoulos, CD Zahiu, AM Zagrean

**Affiliations:** * “Carol Davila” University of Medicine and Pharmacy, Division of Physiology & Fundamental Neuroscience, Bucharest, Romania

**Keywords:** melatonin, embryo-fetal development, circadian rhythm

## Abstract

Melatonin is an indoleamine produced by the pineal gland and secreted in a circadian manner. In the past few decades, research over this topic has been enhanced. Melatonin has many important roles in the human physiology: regulator of the circadian rhythms, sleep inducer, antioxidant, anticarcinogenic. This paper reviews the involvement of melatonin in embryo fetal development. The pineal gland develops completely postpartum, so both the embryo and the fetus are dependent on the maternal melatonin provided transplacentally. Melatonin appears to be involved in the normal outcome of pregnancy beginning with the oocyte quality and finishing with the parturition. Its pregnancy night-time concentrations increase after 24 weeks of gestation, with significantly high levels after 32 weeks. Melatonin receptors are widespread in the embryo and fetus since early stages. There is solid evidence that melatonin is neuroprotective and has a positive effect on the outcome of the compromised pregnancies. In addition, chronodisruption leads to a reproductive dysfunction. Thus, the influence of melatonin on the developing human fetus may not be limited to the entertaining of circadian rhythmicity, but further studies are needed.

## Introduction

Melatonin (N-acetyl-5-methoxytryptamine) is an indoleamine mainly produced by the pineal gland, with numerous important physiological functions, acting as an antioxidant, free radical scavenger, anti-inflammatory, anticarcinogenic, sleep inducer and regulator of the circadian rhythm in the body [**1**].

The circadian system master clock is located in the suprachiasmatic nucleus (SCN) of the hypothalamus, but has also peripherally located biological clocks, which are found in most tissues. This system has an endogenous rhythmicity of approximately 24 hours. Peripheral systems are synchronized by SCN. Melatonin represents the key linkage-molecule between the SCN and the peripheral biological clocks and it is produced and secreted in a circadian fashion, its secretion being stimulated by darkness and inhibited by light [**2**].

In humans, the hormone secretion increases soon after the onset of darkness, peaks in the middle of the night, between 2 and 4 a.m., and progressively falls during the second half of the night [**3**]. The purpose of the current paper is to review the roles of melatonin in embryonic and fetal development up to date. The role of melatonin in pregnancy and embryo fetal development has hardly been discussed, but there is clear evidence of a strong connection between fetal normal development and melatonin.

First, the embryo and fetus are dependent on maternal melatonin, as the pineal gland becomes mature after birth. Melatonin crosses all physiological barriers without being modified, including the placental one [**4**] and has been involved in placental function in animals and human [**5**]. Second, in humans, SCN expresses melatonin receptors, both in adult and fetus. Maternal melatonin enters the fetal circulation transplacentally providing photoperiodic information to the fetus and by that influencing the internal rhythms of the offspring.

Melatonin concentrations increase in maternal blood during pregnancy, reaching a maximum at term. The presence of melatonin has also been demonstrated in amniotic fluid [**6**].

Chronodisruption leads to reproductive dysfunction and appears to be a key contributor to offspring diseases that develop in adult life (the concept of fetal programming). Melatonin decreases in conditions associated with serious outcome for the fetus and seems to be involved in preeclampsia and intrauterine growth restriction [**7**]. Melatonin treatment during human normal or abnormal pregnancy has been studied for a large range of conditions and at different times during the gestational period. Considering the ethical issues, it is more difficult to study a normally occurring pregnancy, than an in vitro fertilization (IVF) one. Melatonin administration started prior to IVF-cycles, continued during pregnancy and was associated with improved pregnancy outcomes [**8**]. Melatonin receptors are widespread in the human fetus from early fetal development. In addition, it appears that the fetuses’ sleep patterns develop in the late pregnancy, melatonin being the regulating factor. A normal sleep pattern is involved in the neurodevelopment and there is solid evidence that melatonin is involved in fetal neuroprotection [**9**]. Thus, the influence of melatonin on the developing human fetus may not be limited to entertaining the circadian rhythmicity.

**Pineal gland development and maturation**

In lower vertebrates, melatonin secretion starts early, during the embryonic period of development. The human fetus or newborn does not produce his own melatonin and is dependent on the hormone supplied by the mother via the placenta and milk. Circadian functions in newborn full-term human infants (melatonin secretion, sleep-wake rhythm, and body temperature rhythm), do not exhibit circadian variation until postnatal 9–12 weeks, and, preterm babies display an important delay in the maturation of pineal function and rhythmic melatonin production.

**Dynamics of melatonin secretion during normal or pathological pregnancies**

Serum melatonin concentrations exhibit changes both in physiological and pathological pregnancies compared to non-pregnant controls. Moreover, melatonin titres are not constant during the 40 weeks of pregnancy, but they show specific dynamics (**Table 1**) [**10**].

**Table 1:** Serum melatonin in pregnant and non-pregnant women

Serum melatonin in the first semester (pmol/l)	29.7 +/- 9.9
Serum melatonin in the second semester (pmol/l)	39.1 +/- 11.2
Serum melatonin in the third semester (pmol/l)	76.5 +/- 38.3
Non-pregnant(pmol/l)	41.7 +/- 15.5

Melatonin concentration was measured in dynamics in normal single fetus pregnant women and in pregnant women with twins, preeclampsia or intrauterine growth retardation and the measurements showed that daytime serum melatonin levels in normal single fetus pregnancies were lower. Also, night-time serum melatonin levels increased after 24 weeks of gestation, with significantly high levels after 32 weeks; these values decreased to non-pregnant levels on the 2nd day postpartum. Night-time serum melatonin levels were significantly higher in twin pregnancies after 28 weeks of gestation than in singleton pregnancies, whereas the patients with severe preeclampsia showed lower serum melatonin levels than the mild preeclampsia or the normal pregnant group after 32 weeks of gestation [**11**].

**Embryonic and fetal expression of melatonin receptors**

The older literature on melatonin describes two major classes of receptors: ML1 (high affinity) and ML2 (low affinity) receptors. There are also at least 3 subtypes of ML1 receptors: Mel1a, Mel1b, and Mel1c. To date, Mel1c receptors appear to be lacking in mammals. Mel1a and Mel1b receptors are now named MT1 and MT2 receptors, respectively. These receptors are classic G-protein-linked receptors that inhibit adenylate cyclase [**12**].

Melatonin receptors have been identified in embryo and fetus, both in the nervous system and peripheral organs, especially in the endocrine system. The presence of 2-[125I]iodomelatonin binding sites in the developing of the human fetal kidney has been detected, correlated with the expression of the mRNA for the G protein-coupled melatonin receptors, Mel1a and Mel1b. Specific 2-[125I]iodomelatonin binding sites were located at the outer periphery of the developing kidney cortex in the nephrogenic region, consisting of differentiating and developing nephrons [**13**]. Another study localized specific, guanosine triphosphate (GTP) sensitive, binding sites of 2-[(125)I]iodomelatonin in the leptomeninges, cerebellum, thalamus, hypothalamus, and brainstem. In the hypothalamus, a specific binding was present in the SCN, as well as the arcuate, ventromedial and mammillary nuclei. In the brainstem, a specific binding was present in the cranial nerve nuclei including the oculomotor nuclei, the trochlear nuclei, the motor and sensory trigeminal nuclei, the facial nuclei, and the cochlear nuclei [**14**].

In Siberian hamsters, melatonin-binding sites were first apparent in the gestational day (GD) 10 over the primitive oral pharynx. From GD 12 to 14, the binding was present over the nasal pharynx, Rathke's pouch, caudal arteries, and over the thyroid gland during its migration along the thyroglossal duct. By GD 16, Rathke's pouch had differentiated into the pituitary gland, which continued to express specific [125I]MEL binding until birth. From GD 16 until birth, the binding was no longer detectable over the thyroid gland, but persisted over the nasal epithelium [**15**].

The presence of specific melatonin binding sites was demonstrated in the middle fetal sheep cerebral artery, brown adipose tissue and adrenal gland. Melatonin concentrations in the nanomolar range or lower have direct inhibitory effects in the middle fetal sheep cerebral arteries and the fetal sheep brown adipose tissue response to noradrenalin, and also in the fetal sheep adrenal gland response to ACTH [**16**].

Melatonin receptors were also identified in the pituitary gland and the median eminence. The pituitary concentration of [125I]melatonin binding sites was highest in the 20-day-old fetuses and then it gradually decreased in the course of postnatal development, until it reached 10% of that value in 29-day-old males. In contrast, the concentration of melatonin receptors in median eminence did not change markedly in the course of development [**17**].

**Melatonin role in normal pregnancy and development**

Melatonin research showed that it plays an important role in pregnancy and parturition. The passage of maternal melatonin through the placenta exposes the fetus to a daily melatonin rhythm of low concentrations during the day and high concentrations at night. Therefore, first, melatonin is obviously involved in inducing a circadian manner of functioning in fetal organs.

The ability of melatonin to promote embryo development in different species has been reported. When mouse embryos were cultured in a medium containing melatonin, increased blastocyst development rates were observed [**18**]. Melatonin has a beneficial role in the in vitro development of rodent embryos found in the 2-cell stage [**19**] and helped the maturation of ovine blastocysts [**20**].

Suppression of maternal plasma melatonin circadian rhythm by continuous light exposure during the second half of gestation showed several effects on fetal development. First, it induced intrauterine growth retardation. Second, in the fetal adrenal in vivo it markedly affected the mRNA expression level of the clock genes and clock-controlled genes, as well as it lowered the content and modified the rhythm of corticosterone. Third, an altered in vitro fetal adrenal response to ACTH of both, corticosterone production and relative expression of clock genes and steroidogenic genes was observed. All these changes were reversed when the mother received a daily dose of melatonin during the subjective night [**21**].

Torres-Farfan et al. reported that the maternal melatonin decreased cortisol production in the fetal adrenal gland of the capuchin monkey [**22**]. In another study on sheep, they found that melatonin had direct inhibitory effects on the noradrenalin-stimulated fetal cerebral artery contraction, the release of glycerol by brown adipose tissue, and on ACTH-induced secretion of cortisol by the fetal adrenal gland. Low levels or a lack of a circadian rhythm of the fetal corticosterone may be the cause of the intrauterine growth retardation that has been previously reported.

The lack of maternal melatonin (induced by pinealectomy) during the early stages of gestation was found to disrupt drinking behavior of rat pups, an effect reversed by the administration of exogenous melatonin to the dam [**23**]. Melatonin is important in normal placental development and function, a function supported by the placenta melatonin receptors expression during early pregnancy [**24**].

**Chronodisruption leads to reproductive dysfunction**

Women working an evening shift or a night shift, or who are transmeridian travelling, report an increased or decreased menstrual cycle length, changes in the duration and amount of menstrual bleeding and dysmenorrhea [**25**]. These symptoms are not subjective, as they correlate with changes in the hormonal profile of ovarian and pituitary origin, such as a high follicular stimulating hormone (FSH) and prolonged follicular stage of the ovarian cycle [**26**].

The working environment also affects the pregnancy outcomes. The shift work while pregnant is also associated with a high risk of prematurity and/ or low for gestational age babies, spontaneous abortion and subfecundity [**27**]. Shift work and jet lag effects on health may be explained by the secondary reduction of the total amount of sleep. FSH concentration in women who sleep less than 8 h/night lowers with 1/5 compared to women with longer sleep duration [**28**]. Total or partial sleep deprivation increases LH amplitude, estradiol and FSH concentrations in normal cycling women and increased estrogen is associated with a high risk of breast cancer [**29**].

**Melatonin and IVF**

One important cause of female infertility is poor oocyte quality. Reactive oxygen species (ROS) are normally produced within the ovarian follicle, during ovulation, and an increased production may be a cause of impaired oocyte maturation. Considering its well established role in scavenging free radicals, melatonin treatment during human pregnancy may help lower the high oxidative stress and may be a possible treatment in some forms of infertility. Melatonin has been studied in assisted reproductive technology, aiming to improve the oocyte quality and pregnancy rates following in vitro fertilization (IVF). Melatonin administration, started before IVF-cycles and continued during pregnancy, was associated with improved pregnancy outcomes. Fertilization success and pregnancy rate were improved by melatonin treatment. Fertilization rate was 50% higher in melatonin treatment cycle compared to the previous melatonin-free cycle (20.2%) [**30**,**31**].

In 2012, Unfer et al. have reported in a personal communication that all the babies born from melatonin-treated mothers were healthy and with no congenital abnormalities. More than that, maternal melatonin treatment significantly improves placental antioxidant enzyme gene expression [**32**]. No maternal and/ or embryo-fetal toxicity effects, due to melatonin treatment, have ever been reported. A median lethal dose in mice could not even be established because an increased mortality rate was not observed, even following the administration of extremely high doses of up to 800 mg/ kg melatonin [**33**].

**Melatonin and the ovarian function**

The role of melatonin in the production of female gametes focused on its direct actions in the ovary. Melatonin specifically concentrates in the ovary when injected systemically [**34**]. Studies have shown that high levels of melatonin are found in human preovulatory follicular fluid in concentrations that are much higher than those in serum [**35**].

It has been reported that the follicular fluid melatonin levels depend directly proportional on the follicular growth. These findings are probably linked to the high levels of ROS produced during the follicular maturation and the antioxidative properties of melatonin, but further investigation is needed.

**Melatonin and fetal neuroprotection**

Melatonin has a neuroprotective effect both in adult and fetal brain. In adults, the most important poof is its positive effect in the treatment of neurodegenerative diseases like Alzheimer or Parkinson disease [**36**]. Neuroprotection efficacy of melatonin in the fetal and neonatal brain was reported in many animal studies. Melatonin was given 10 minutes after the hypoxic acidemia episode in the fetal sheep and decreased the incidence of cell death and the number of activated microglial cells present in the brain [**37**]. The administration of melatonin at the time of induced hypoxia in the fetal sheep brain also reduced the inflammation and cell death.

In a recent study, melatonin treatment before and during transient severe fetal asphyxia lowered oxidative stress and stopped the formation of hydroxyl radicals within the fetal brain, reduced lipid peroxidation and cell death and stabilized the blood–brain barrier [**38**].

Melatonin maternal administration of melatonin reduced the fetal hypoxia in an animal model of fetal growth restriction, improved neurodevelopment and decreased brain injury and oxidative stress in newborn lambs [**39**]. The neuroprotective potential of melatonin is sustained by the fact that melatonin inhibits middle cerebral artery constriction produced by norepinephrine [**40**] and induces umbilical vasodilation in ovine models [**41**].

In a small clinical trial, melatonin has been orally administrated to newborn babies with birth asphyxia, and was shown to significantly reduce oxidative stress by decreasing plasma levels of malondialdehyde and nitrate/ nitrite. This study holds very strong evidence of melatonin efficiency as 3 of 10 asphyxiated babies died in the non-melatonin treated group, while no deaths were recorded in the melatonin treated group [**42**].

**Fetal programming concept and melatonin**

Fetal programming is an emerging concept that links environmental conditions during embryonic and fetal development with risk of diseases later in life. Compromised pregnancies (gestational diabetes mellitus, intrauterine growth retardation, preeclampsia, maternal under-nutrition, and maternal stress) may affect fetal development. The pathophysiological basis of this outcome may be the induction of high oxidative stress in these pregnancies, which secondary alters fetal development. Melatonin night levels in pregnant women with preeclampsia are significantly lower compared with normal pregnancies (48.4 ± 24.7 vs. 85.4 ± 26.9 pg/mL). Circadian blood pressure and melatonin secretion rhythm followed a parallel course in preeclampsia, both during pregnancy and, at least 2 months after delivery [**43**].

Another theory is the existence of epigenetic inheritance system, through DNA superstructure changes occurring as a response to pregnancy environmental conditions. It is believed that melatonin is the key regulator of this kind of transmission and that it acts through nuclear melatonin receptors, producing DNA bending in the oocyte [**44**].

## Conclusions

Melatonin is an important regulator of the complex embryo-fetal developmental processes. First, it induces the circadian rhythmicity in the offspring. Second, it appears to have a direct developmental effect on nervous and endocrine system. It also protects the highly metabolic ROS producing organs in the development from the oxidative stress damage.

Melatonin is currently an over-the-counter drug, with a high availability and apparently without acute adverse effects. Even though there are a few clinical studies on pregnant women that show melatonin as being risk-free, we consider that regarding the extensive and yet not known effects on development, it should not be used by pregnant women before further studies.

It has been shown that melatonin has neuroprotective effects in animal models. Considering the newly investigated key-role of melatonin as an epigenetic transducer, further studies are required to confirm that there are no late onset adverse reactions in adult life.

**Sources of funding**

Founding through POSDRU/107/1.5/S/82839.

**Disclosures**

None

## References

[1] Reiter RJ, Tan DX, Galano A (2014). Melatonin: Exceeding Expectations Physiology (Bethesda).

[2] Cajochen C, Krauchi K, Wirz-Justice A (2003). Role of Melatonin in the Regulation of Human Circadian Rhythms and Sleep. Journal of Neuroendocrinology.

[3] Lynch HJ, Wurtman RJ, Moskowitz MA, Archer MC, Ho MH (1975). Daily rhythm in human urinary melatonin. Science.

[4] Okatani Y, Okamoto K, Hayashi K, Wakatsuki A, Tamura S, Sagara Y (1998). Maternal–fetal transfer of melatonin in pregnant women near term. J Pineal Res.

[5] Kennaway DJ (2000). Melatonin and development: physiology and pharmacology. Semin Perinatol.

[6] Kivela A, Kauppila A, Leppaluoto J, Vakkuri O (1989). Serum and amniotic fluid melatonin during human labor. J Clin Endocrinol Metab.

[7] Nakamura Y, Tamura H, Kashida S, Takayama H, Yamagata Y, Karube A, Sugino N, Kato H (2001). Changes of serum melatonin level and its relationship to feto-placental unit during pregnancy. J Pineal Res.

[8] Rizzo P, Raffone E, Benedetto V (2010). Effect of the treatment with myo-inositol plus folic acid plus melatonin in comparison with a treatment with myo-inositol plus folic acid on oocyte quality and pregnancy outcome in IVF cycles. A prospective, clinical trial. Eur Rev Med Pharmacol Sci.

[9] Gitto E, Marseglia L, Manti S, D’Angelo G, Barberi I, Salpietro C, Reiter RJ (2013). Protective Role of Melatonin in Neonatal Diseases. Oxidative Medicine and Cellular Longevity.

[10] Kivelä A (1991). Serum melatonin during human pregnancy. Acta Endocrinol (Copenh).

[11] Naamura Y (2001). Changes of serum melatonin level and its relationship to feto-placental unit during pregnancy. Journal of Pineal Research.

[12] Zlotos DP, Jockers R, Cecon E, Rivara S, Witt-Enderby PA (2014). MT1 and MT2 Melatonin Receptors: Ligands, Models, Oligomers, and Therapeutic Potential. J. Med. Chem.

[13] Drew JE, Williams LM, Hannah LT, Barrett P, Abramovich DR (1998). Melatonin receptors in the human fetal kidney: 2-[125I]iodomelatonin binding sites correlated with expression of Mel1a and Mel1b receptor genes. J Endocrinol.

[14] Thomas L, Purvis CC, Drew JE, Abramovich DR, Williams LM (2002). Melatonin receptors in human fetal brain: 2-[(125)I]iodomelatonin binding and MT1 gene expression. J Pineal Res.

[15] Rivkees SA, Reppert SM (1991). Appearance of melatonin receptors during embryonic life in Siberian hamsters (Phodopus sungorous). Brain Research.

[16] Torres-Farfan C, Valenzuela FJ, Mondaca M, Valenzuela GJ, Krause B, Herrera EA, Riquelme R, Llanos AJ, Seron-Ferre M (2008). Evidence of a role for melatonin in fetal sheep physiology: direct actions of melatonin on fetal cerebral artery, brown adipose tissue and adrenal gland. The Journal of Physiology.

[17] Vaněček J (1988). The Melatonin Receptors in Rat Ontogenesis. Neuroendocrinology.

[18] Ishizuka B, Kuribayashi Y, Murai K, Amemiya A, Itoh MT (2000). The effect of melatonin on in vitro fertilization and embryo development in mice. J Pineal Res.

[19] Tian XZ, Wen Q, Shi JM, Liang-Wang, Zeng SM, Tian JH, Zhou GB, Zhu SE, Liu GS (2010). Effects of melatonin on in vitro development of mouse two-cell embryos cultured in HTF medium. Endocr Res.

[20] Sampaio RV, Conceição DSB, Moysés SM, Sampaio LFS, Ohashi OM (2012). MT3 melatonin binding site, MT1 and MT2 melatonin receptors are present in oocyte, but only MT1 is present in bovine blastocyst produced in vitro. Reproductive Biology and Endocrinology.

[21] Mendez N, Abarzua-Catalan L, Vilches N, Galdames HA, Spichiger C, Richter HG, Valenzuela GJ, Seron-Ferre M, Torres-Farfan C (2012). Timed Maternal Melatonin Treatment Reverses Circadian Disruption of the Fetal Adrenal Clock Imposed by Exposure to Constant Light. PLoS One.

[22] Torres-Farfan C, Richter HG, Germain AM, Valenzuela GJ, Campino C, Rojas-García P, Forcelledo ML, Torrealba F, Serón-Ferré M (2004). Maternal melatonin selectively inhibits cortisol production in the primate fetal adrenal gland. J Physiol.

[23] Kennaway DJ, Stamp GE, Goble FC (1992). Development of melatonin production in infants and the impact of prematurity. J Clin. Endocrinol Metab.

[24] Tamura H, Nakamura Y, Terron MP, Flores LJ, Manchester LC, Tan DX, Sugino N, Reiter RJ (2008). Melatonin and pregnancy in the human. Reprod. Toxicol.

[25] Labyak S, Lava S, Turek F, Zee P (2002). Effects of shift work on sleep and menstrual function in nurses. Health Care for Women International.

[26] Knutsson A (2003). Health disorders of shift workers. Occupational Medicine.

[26'] Chung FF, Yao CCC, Wan GH (2005). The associations between menstrual function and life style/working conditions among nurses in Taiwan. J Occupational Health.

[27] Knutsson A (2003). Health disorders of shift workers. Occupational Medicine.

[28] Davis S, Mirick DK, Chen C, Stanczyk FZ (2012). Night Shift Work and Hormone Levels in Women Cancer. Epidemiol Biomarkers Prev.

[29] Baumgartner A, Dietzel M, Saletu B (1993). Influence of partial sleep deprivation on the secretion of thyrotropin, thyroid hormones, growth hormone, prolactin, luteinizing hormone, follicle stimulating hormone, and estradiol in healthy young women. PsychiatryResearch.

[30] Unfer V, Raffone E, Rizzo P (2011). Effect of a supplementation with myo-inositol plus melatonin on oocyte quality in women who failed to conceive in previous in vitro fertilization cycles for poor oocyte quality: a prospective, longitudinal, cohort study. Gynecol Endocrinol.

[31] Tamura H, Nakamura Y, Terron MP (2008). Melatonin and pregnancy in the human. Reprod Toxicol.

[32] Okatani Y, Okamoto K, Hayashi K (1998). Maternal-fetal transfer of melatonin in pregnant women near term. J Pineal Res.

[33] Barchas J, Dacosta F, Spector S (1967). Acute pharmacology of melatonin. Nature.

[34] Wurtman RJ, Axelrod J, Potter LT (1964). The uptake of H3-melatonin in endocrine and nervous tissues and the effects of constant light exposure. J Pharmacol Exp Ther.

[35] Brzezinski A, Seibel MM, Lynch HJ, Deng MH, Wurtman RJ (1987). Melatonin in human preovulatory follicular fluid. J Clin Endocrinol Metab.

[36] Olakowska E, Marcol W, Kotulska K, Lewin-Kowalik (2005). The role of melatonin in the neurodegenerative diseases. J Bratisl Lek Listy.

[37] Welin AK, Svedin P, Lapatto R (2007). Melatonin reduces inflammation and cell death in white matter in the mid-gestation fetal sheep following umbilical cord occlusion. Pediatr Res.

[38] Yawno T, Castillo-Melendez M, Jenkin G (2012). Mechanisms of melatonin-induced protection in the brain of late gestation fetal sheep in response to hypoxia. Dev Neurosci.

[39] Supramaniam VG, Jenkin G, Loose J (2006). Chronic fetal hypoxia increases activin A concentrations in the late-pregnant sheep. BJOG Int J Obstet Gynaecol.

[40] Thakor AS, Herrera EA, Serón Ferré M (2010). Melatonin and vitamin C increase umbilical blood flow via nitric oxide dependent mechanisms. J Pineal Res.

[41] Miller SL, Yan EB, Castillo-Melendez M (2005). Melatonin provides neuroprotection in the late-gestation fetal sheep brain in response to umbilical cord occlusion. Dev Neurosci.

[42] Fulia F, Gitto E, Cuzzocrea S, Reiter RJ, Dugo L, Gitto P (2001). Increased levels of malondialdehyde and nitrite/nitrate in the blood of asphyxiated newborns: reduction by melatonin. J. Pineal Res.

[43] Bouchlariotou S, Liakopoulos V, Giannopoulou M, Arampatzis S, Eleftheriadis T, Mertens PR, Zintzaras E, Messinis IE, Stefanidis I (2014). Melatonin secretion is impaired in women with preeclampsia and abnormal circadian blood pressure rhythm. Renal Failure.

[44] Irmak MK, Topal T, Oter S (2005). Melatonin seems to be a mediator that transfers the environmental stimuli to oocytes for inheritance of adaptive changes through epigenetic inheritance system. Medical Hypotheses.

